# Resistance pattern of *Escherichia coli* to levofloxacin in Iran: a narrative review

**Published:** 2020-06

**Authors:** Gholamhossein Hassanshahi, Ali Darehkordi, Mahmood Sheikh Fathollahi, Soudeh Khanamani Falahati-Pour, Ebrahim Rezazadeh Zarandi, Shokrollah Assar

**Affiliations:** 1 Molecular Medicine Research Center, Research Institute of Basic Medical Sciences, Rafsanjan University of Medical Sciences, Rafsanjan, Iran; 2 Department of Chemistry and Biophysics, School of Sciences, Vali-e-Asr University of Rafsanjan, Rafsanjan, Iran; 3 Department of Social Medicine, School of Medicine, Rafsanjan University of Medical Sciences, Rafsanjan, Iran; 4 Pistachio Safety Research Centre, School of Medicine, Rafsanjan University of Medical Sciences, Rafsanjan, Iran; 5 Department of Microbiology, School of Medicine, Rafsanjan University of Medical Sciences, Rafsanjan, Iran

**Keywords:** *Escherichia coli*, Fluoroquinolones, Levofloxacin, Iran, Antibiotic resistance

## Abstract

Fluoroquinolones (FQs) are widely used in the treatment of infections caused by *Escherichia coli*. FQs are broad spectrum antibiotics with high tissue penetration, and ease of use. Therefore, given the concerns existing about drug resistance, we aim to review the latest findings about resistance patterns to levofloxacin (LVX) along with other FQs in *E. coli* infections in different parts of Iran. Evidence shows that quinolones have been used in Iran for nearly 50 years, and that 0–65% of *E. coli* isolates show resistance to FQs. In the western parts of Iran, the highest rate of resistance to LVX (66.7%) has been reported among patients having urinary tract infections with *E. coli* isolates. Few studies and information exist on the antimicrobial resistance of *E. coli* to LVX in different geographical locations of Iran. However, the findings of various studies on this subject show that *E. coli* resistance to LVX is more in the western part of Iran than in central and southern regions, but it is similar among inpatients and outpatients. Therefore, it is reasonable advisable to limit the overuse, inappropriate prescription, and self-medication of LVX to prevent the induction of FQ-resistant strains. Accordingly, in order to obtain a clearer image of resistance to FQs, especially LVX in *E. coli* in Iran, more extensive investigations in different geographical locations and periods of time are required. In addition, antimicrobial stewardship would be helpful in this regard.

## INTRODUCTION

Since the discovery and synthesis of antibiotics, life expectancy increased by 10 years; however, the World Health Organization (WHO) reported antibiotic resistance as a “major global threat” in 2014. According to a report by the UN-affiliated agency on 30^th^ April 2014, the world is entering the “post-antibiotic” period when simple infections that were curable for years would threaten individuals’ life. The therapeutic effects of antibiotics are on the decline at an alarming and inevitable pace similar to the phenomenon of global warming (1, 2). Antimicrobial resistance is greatly increasing among bacteria, and governments are spending large amounts of budgets to control it. It is necessary to use appropriate drugs for the treatment of infections (3, 4). *Escherichia coli* is a bacterium in which antimicrobial resistance is increasingly high (5).

This microorganism is normally found in the gut microbiomes of mammals, birds, and fish (6, 7). It provides some benefits to the hosts and plays an important role in the production of vitamins K and B12 both in hosts and biotechnology (7, 8). However, *E. coli* is a major human pathogen that causes urinary tract infections (UTIs), enterocolitis, and septicemia in humans. Over 10 pathotypes of *E. coli* have been identified to date, and different pathotypes could cause similar diseases through various mechanisms (5, 9).

Nalidixic acid was the first quinolone synthesized in 1962 (10). Due to its low oral absorption, limited antibacterial spectrum, high ability to bind to proteins, and low tolerability, nalidixic acid was restricted in treating bacterial infections. Thus, many fluoroquinolone analogs have been produced since the last decades (11). For example, norfloxacin, ciprofloxacin, and LVX were patented in 1987, 1980, and 1965, respectively (12, 13).

LVX, the L-isomer of ofloxacin and one of the FQs, has been available since 1992 (14, 15). Following the introduction of LVX and due to its strong effects on a wide range of Gram-positive and Gram-negative bacteria, many studies have been conducted to determine its benefits. However, researchers have reported that LVX overuse gradually induces the expression of resistant genes and leads to the emergence of resistant strains. Therefore, standardized discs containing LVX have been introduced, with the susceptibility patterns of different bacteria evaluated (16–18). LVX interferes with the DNA replication of the bacterium by inhibiting the activity of the DNA gyrase enzyme, thereby inhibiting the DNA synthesis of the bacterium. This characteristic prevents bacterial replication and produces bactericidal effects (19).

LVX is frequently prescribed to treat *E. coli* infections (14, 20–22). Because LVX is an effective therapeutic agent, it is prescribed by Iranian physicians for treating infections. It has some properties, including daily administration, high effectiveness, low cost, and minimum side effects (23). Several studies have been conducted on the antimicrobial resistance of *E. coli* to many antibiotics in Iran; however, few studies have been carried out to determine its resistance to LVX. Moreover, in a recent study on the limitation of the use of LVX, a reduction in the frequency of FQ-resistant *E. coli* species has been reported (24). Therefore, the present review aims to achieve a clearer image of the antimicrobial resistance of *E. coli* to LVX in Iran.

### The mechanism of FQs’ function.

Quinolones with fluorine in their structure are called FQs, and LVX is a member of fluoroquinolone antibiotics. Fluorination enhances the effects of quinolones on both Gram-negative and Gram-positive bacteria and improves drug entry into the bacterial cells (25). According to the antimicrobial activity spectrum, quinolones are classified into four generations; the first generation includes cinoxacin, pefloxacin, and nalidixic acid; the second generation consists of ofloxacin, ciprofloxacin, enoxacin, lomefloxacin, and norfloxacin; the third generation is comprised of gemifloxacin, LVX, sparfloxacin, gatifloxacin, and moxifloxacin; and the fourth generation includes trovafloxacin and alatrofloxacin (10).

Quinolones have impacts on some facultative anaerobic Gram-negative bacilli, such as enterobacteriaceae. All compounds from the quinolone family have approximately a similar basic structure (4-aminoquinoline) and a mechanism of action (26). They inhibit DNA replication both selectively and reversibly, and stop sensitive bacteria by limiting the function of DNA gyrase and topoisomerase IV. DNA gyrase is a tetrameric enzyme with two subunits of A and B. These two subunits are encoded by *gyrA* and *gyrB* genes and play a central role in the unwinding and twisting processes of DNA. Similar to DNA gyrase, topoisomerase IV is a tetrameric enzyme with two subunits. These subunits are encoded by *parE* and *parC* genes and involved in separating two daughter chromosomes during the replication process. FQs commonly interfere with the GyrA subunit of DNA gyrase (27).

FQs serve as bactericidal drugs by inhibiting DNA replication and transcription. Both DNA strands are interconnected within the bacterial cell. For bacterial division, two strands of DNA should be separated from each other. To facilitate the separation of the strands, DNA gyrase helps strands get detached and rejoined (28, 29). Thus, DNA gyrase is really essential for the survival and proliferation of bacteria. Most eukaryotic cells, especially in humans, lack this enzyme. Therefore, it could be considered an attractive target for antibacterial compounds, especially quinolones (30).

### lvx’s antimicrobial activity.

LVX is most often used in treating patients suffering from several disorders, such as arthritis and skin infections, as well as disorders in the lungs, airways, sinuses, bones, urethra, kidneys, and prostate (31). It is reactive against a broad spectrum of bacteria and affects both Gram-positive and Gram-negative aerobic bacteria as well as some drug-resistant anaerobes. In addition, LVX is highly effective in treating unusual bacterial infections, such as non-gonococcal urethritis caused by *Chlamydia trachomatis, Mycoplasma genitalium, Ureaplasma urealyticum, E. coli*-induced diarrhea, *Campylobacter jejuni* and *Shigella* spp. (32).

### *E. coli* resistance to lvx in the world.

*E. coli* resistance to FQs was very low at approximately less than 2% in the 1990s; however, its resistance to LVX has significantly increased by more than 50% in 2000 (33). The resistance of some extended-spectrum beta-lactamase (ESBL)-producing microorganisms to LVX has reached 100%. In terms of geography, the reported prevalence rate of resistance to FQs varies among the European countries, from 7.9% in Sweden to 52% in Turkey. In the Asian countries, the prevalence rate of FQ-resistant *E. coli* in the samples of community-acquired UTI was reported to be about 25% in Korea (34) and 69% in India (35). In the United States, the frequency of LVX-resistant *E. coli* was 1% in 1994, but it varied from 2 to 27% among out-patients and inpatients with UTI in 2014 (36). It was reported that 23.4% of *E. coli* isolates in the Japanese population were resistant to LVX in 2012 (37).

It has been verified that *E. coli* resistance has been remarkably increasing to FQs, such as LVX, throughout the world. Within the 4-year period of 2005–2009, *E. coli* resistance to LVX increased from 29.49 to 43.20%. However, the resistance pattern of *E. coli* differed from one year (43.20% in 2008) to another (31.75% in 2009) (38). Studies conducted on the antimicrobial resistance of *E. coli* in North American countries in addition to the high resistance of *E. coli* to LVX suggest that at least it would be preferable not to choose LVX as the first line of treatment in complicated UTIs (39).

### *E. coli* resistance to FQs and lvx in Iran.

Many factors are involved in the induction of antibiotic resistance in *E. coli* (40), which are different in developed, developing, and underdeveloped countries (41). Most of the studies in this field have been carried out in developed countries, so the comparison of bacterial resistance patterns to other countries, such as Iran, is relatively difficult (40). Thus, the comparison of the LVX resistance pattern of *E. coli* inside different geographical regions of Iran to other countries could be useful.

FQs have been prescribed in Iran for about 50 years. Several studies have been conducted in recent years on the sensitivity and resistance patterns of *E. coli* to LVX and other FQs (Table 1). One of the first studies that investigated quinolone resistance in Iran was carried out by Gharegozlou et al. (1968). They studied 1,251 urine specimens and found that *E.coli* (131 samples) was the most frequent bacterium in urine cultures. In their studies, *E. coli* was shown to be sensitive to nalidixic acid in 16 out of 20 samples (80%) (42).

**Table 1.** Fluoroquinolones resistance pattern of *E. coli*

**No.**	**Sample Type**	**In- or Out-Patient**	**City**	**Sample size**	**Type of Quinolone**	**Antibiogram Result (%)**			**Author**	**Study Year**	**Publish Year**	**Reference**

						**S**	**I**	**R**				
1	Respiratory	In	Tehran	48	Levofloxacin	70.8	16.6	12.5	Haeili	2009–2011	2013	43
				(ESBL^*^=10) (non ESBL=38)	Ciprofloxacin	70.8	0	29.1				
2	Stool	In	Shiraz	54	Levofloxacin	NR	NR	5.56	Ghorbani-Dalini	2010	2015	52
				(ESBL=7) (non ESBL=47)	Ciprofloxacin	NR	NR	8.33				
3	Stool	Out	Shiraz	8 (DAEC)	Levofloxacin	87.5	12.5	0	Abbasi	2012–2013	2016	51
					Ciprofloxacin	75	25	0				
					Nalidixic acid	37.5	25	37.5				
4	Stool	Out	Tehran	31 (ESBL)	Levofloxacin	NR	NR	3.2	Kazemian	2014–2015	2016	53
					Ciprofloxacin	NR	NR	29.3				
				19 (non ESBL)	Levofloxacin	NR	NR	0				
					Ciprofloxacin	NR	NR	3.2				
5	Urine	In	Khorramabad	140	Levofloxacin	57.1	NR	42.9	Firoozeh	2012	2014	46
					Ciprofloxacin	55	NR	45				
					Nalidixic acid	82.8	NR	17.2				
					Norfloxacin	52.9	NR	47.1				
					Ofloxacin	55	NR	45				
6	Urine	Out	Estahban	224	Levofloxacin	70.5	5.8	23.7	Rashki	2012–2013	2015	48
					Ciprofloxacin	56.3	4.5	39.2				
					Nalidixic acid	44.6	6.7	48.7				
					Norfloxacin	67.4	4.9	27.7				
					Ofloxacin	68.3	2.7	29				
7	Urine	In	Zanjan-Qazvin	200	Levofloxacin	42.5	1.5	56	Rezazadeh	2014–2015	2016	47
					Ciprofloxacin	44	0	56				
					Nalidixic acid	32.5	1	66.5	Akya	2014–2015	2017	44
					Norfloxacin	44.5	0	55.5				
					Gatifloxacin	42	0	58				
8	Urine	Out	Kermanshah	66 (ESBL)	Levofloxacin	28.8	4.5	66.7				
					Ciprofloxacin	28.8	6	65.2				
					Nalidixicacid	15.2	0	84.8				
				174 (non ESBL)	Levofloxacin	76.4	2.3	21.3				
					Ciprofloxacin	76.4	3.5	20.1				
					Nalidixicacid	78.7	0	21.3				
9	Urine	Out	Yasuj	144	Levofloxacin	34.7	NR	NR	Mirzaei	2014–2015	2018	56
					Ciprofloxacin	36.1	NR	NR				
					Nalidixicacid	60.4	NR	NR				
					Gatifloxacin	35.4	NR	NR				
10	Urine	In	Shiraz	121	Levofloxacin	49.6	2.5	47.9	Malekzadegan	2016–2017	2019	50
					Ciprofloxacin	41.3	9.9	48.4				
					Nalidixic acid	19	9.1	71.9				
					Norfloxacin	44.6	4.1	51.2				
					Ofloxacin	46.3	3.3	50.4				
11	Mix^*^	In	Semnan	216	Levofloxacin	38.4	NR	NR	Pajand	2014	2017	54
					Ciprofloxacin	36.1	NR	NR				
12	Mix	In	Tehran	16	Levofloxacin	56.2	NR	43.8	Azimi	2013–2018	2019	55
				49	Ciprofloxacin	53.1	NR	46.9				
				6	Nalidixicacid	83.3	NR	16.7				

Abbreviation: S: Sensitive, I: Intermediate, R: Resistant, ESBL: Extended Spectrum Beta-Lactamases, NR: Not Reported, DAEC: Diffuse-Adhering *Escherichia Coli*, Mix: Mixed clinical samples

**Fig. 1.**
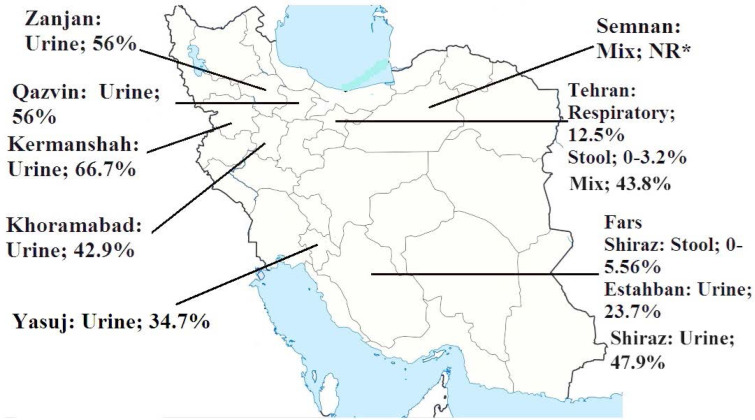
Percentage of levofloxacin resistance *E. coli* in Iran

Iranian studies on the pattern of *E. coli* resistance to LVX have given inconsistent results. The report by Haeli et al. (2013) is the first available Iranian study to have investigated the LVX resistance pattern of *E. coli* in parallel with other FQs. They claimed *E. coli* resistance to LVX was 12.5% in Tehran population in the time period of 2009–2011 (43). The highest rate of resistance to LVX (64.3%) was reported by Akya et al. (2017) in patients with UTI caused by ESBL-producing isolates of *E. coli* in Kermanshah. However, the lowest rate of resistance to LVX was reported in non-ESBL isolates (21.3%). In their study, 66 out of 240 isolates (27.5%) produced ESBLs (44). In another Iranian report, the frequency of ESBL-producing *E. coli* isolates showed to be up to 89% (45). In other studies, resistance to LVX in UTI patients was 21.3–66.7% (44–50). Nonetheless, the proportion of ESBL-producing isolates was determined in these studies. LVX resistance in *E. coli* isolates from patients with diarrhea is surprisingly low. For instance, it was 0% and 5.6% in non-ESBL- and ESBL-producing isolates, respectively (51–53). Two studies used more than one organ specimen (specimens from different body sites), and the rate of resistance to LVX was 43.8% and 61.6% in the hospitalized patients (Fig. 1) (54, 55).

The antimicrobial susceptibility of *E. coli* to FQs, especially LVX, has been more or less examined in Iran. Such studies indicate that *E. coli* strains isolated from UTI exhibit the highest rate of resistance to LVX among other samples. However, LVX resistance was different in various geographic regions of Iran. The antimicrobial resistance of *E. coli* to LVX was higher in the western provinces of Iran, such as Lorestan (46), Qazvin, Zanjan (47), and Kermanshah (44) than in the central ones, such as Tehran, Alborz, and Semnan (54, 55), as well as in the southern province of Fars (51, 52). According to the study conducted in Yasuj, the center of Kohgiluyeh and Boyer-Ahmad Province, 50 (34.7%) of 144 urine samples showed *E. coli* resistance to LVX (56). Four different studies conducted in the southern regions of Iran, i.e. Shiraz and Estahban) (48, 50–52), reported the antimicrobial resistance of *E. coli* isolates to LVX at 5.56 to 47.9%, while it was much higher (42.9–66.7%) in the western regions (44, 46, 47). One of the major points of present study was the significant difference in the resistance rate of *E. coli* isolated from inpatients and outpatients (Table 1). Therefore, one could conclude that the pattern of resistance to LVX in hospitalized and community-acquired patients did not significantly change from 2000 to 2015. The inappropriate administration, excessive prescription, and prescription of LVX without the antibiogram algorithm could be the reasons for not observing a difference between *E. coli* isolates from inpatients and outpatients in the Iranian health system. A study has been conducted in Tehran (the capital of Iran) in different periods of time, which have reported the pattern of *E. coli* resistance to LVX. According to this study, antimicrobial resistance to LVX has fluctuated between 33.3% and 100% during 2013 to 2018 (55). In addition, data from other geographical regions of Iran indicate that LVX resistance has been following an ascendant pattern.

To sum it up, few studies have been conducted in Iran on *E. coli* resistance to FQs, especially LVX. Many studies have reported that the level of *E. coli* resistance to LVX in the western part of Iran has been increasing like many other countries in the world (44, 46, 47). The main reasons for the high prevalence of such resistance could be (a) the use of antibiotics in livestock, (b) the conditions of animal husbandry with livestock being common in the western region of Iran, (c) the local resistance pattern, (d) the duration of antibiotics consumption, (e) excessive prescription of antibiotics, (f) self-medication, (g) low health standards, (h) old age, (i) use of LVX for 48 hours during the last year (57). Indeed, to obtain a clearer view of the local pattern of *E. coli* resistance to LVX, further studies are required with different samples in different geographic regions of Iran to obtain comprehensive and accurate information about *E. coli* resistance to FQs, especially LVX.

## CONCLUSION

It is worth saying that all aforementioned studies have been carried out on samples collected from out-patients and inpatients in different regions. Therefore, it is difficult to make a comparison among them. Most studies were conducted in a short period of time (one year) and in a limited geographical region. According to the literature, although LVX has not been used for a long time in Iran, the resistance of *E. coli*, as a nosocomial pathogen, has been increasingly high due to LVX overuse. Thus, more attention must be paid to employing stewardship antibiotics.

## References

[1] Prasad K Lekshmi G Ostrikov K Lussini V Blinco J Mohandas M Synergic bactericidal effects of reduced graphene oxide and silver nanoparticles against Gram-positive and Gram-negative bacteria. Sci Rep 2017;7: 1591. 28484209 10.1038/s41598-017-01669-5 PMC5431540

[2] Burnham C-AD Leeds J Nordmann P O’Grady J Patel J. Diagnosing antimicrobial resistance. Nat Rev Microbiol 2017; 15: 697–703. 29021600 10.1038/nrmicro.2017.103

[3] Paharik AE Schrebier HL Spaulding CN Dodson KW Hultgren SJ. Narrowing the spectrum: the new frontier of precision antimicrobials. Genome Med 2017; 9: 110. 29241446 10.1186/s13073-017-0504-3 PMC5729598

[4] Eurosurveillance editorial team. European union summary report on antimicrobial resistance in zoonotic and indicator bacteria from humans, animals and food 2012 published. Euro Surveill 2014; 19: 20748. 24698141 10.2807/ese.19.12.20748-en

[5] Allocati N Masulli M Alexeyev MF Di Ilio C. *Escherichia coli* in Europe: an overview. Int J Environ Res Public Health 2013; 10: 6235–6254. 24287850 10.3390/ijerph10126235 PMC3881111

[6] Leimbach A Hacker J Dobrindt U. *E. coli* as an all-rounder: the thin line between commensalism and pathogenicity. Curr Top Microbiol Immunol 2013; 358: 3–32. 23340801 10.1007/82_2012_303

[7] Blount ZD. The unexhausted potential of *E. coli*. eLife 2015; 4: e05826. 10.7554/eLife.05826 PMC4373459 25807083

[8] Baeshen MN Al-Hejin AM Bora RS Ahmed M Ramadanass H Saini KS Production of biopharmaceuticals in *E. coli*: current scenario and future perspectives. J Microbiol Biotechnol 2015; 25: 953–962. 25737124 10.4014/jmb.1412.12079

[9] Miri ST Dashti A Mostaan S Kazemi F Bouzari S. Identification of different *Escherichia coli* pathotypes in north and north-west provinces of Iran. Iran J Microbiol 2017; 9: 33–37. 28775821 PMC5534002

[10] Pham TD Ziora ZM Blaskovich MA. Quinolone antibiotics. Medchemcomm 2019; 10: 1719–1739. 31803393 10.1039/c9md00120d PMC6836748

[11] Darehkordi A Javanmiri M Ghazi S Assar S. Synthesis of N-aryl-2, 2, 2-trifluoroacetimidoyl piperazinylquinolone derivatives and their antibacterial evaluations. J Fluor Chem 2011; 132: 263–268.

[12] Stone MRL Masi M Phetsang W Pagès JM Cooper MA Blaskovich MAT. Fluoroquinolone-derived fluorescent probes for studies of bacterial penetration and efflux. Medchemcomm 2019; 10: 901–906. 31303987 10.1039/c9md00124g PMC6596217

[13] Rehman A Patrick WM Lamont IL. Mechanisms of ciprofloxacin resistance in *Pseudomonas aeruginosa*: new approaches to an old problem. J Med Microbiol 2019; 68: 1–10. 30605076 10.1099/jmm.0.000873

[14] Bernard J Armand-Lefèvre L Luce E El Mniai A Chau F Casalino E Impact of a short exposure to levofloxacin on faecal densities and relative abundance of total and quinolone-resistant *Enterobacteriaceae*. Clin Microbiol Infect 2016; 22: 646.e1–4. 10.1016/j.cmi.2016.04.015 27126608

[15] Gill GK Chhabra M Chawla SP. Levofloxacin-induced desquamation: A possible and rare case report. Curr Drug Saf 2020; 15: 61–64. 31284870 10.2174/1574886314666190708152223

[16] Pan Z Liu R Zhang P Zhou H Fu Y Zhou J. Combination of tigecycline and levofloxacin for successful treatment of nosocomial pneumonia caused by New Delhi Metallo-β-Lactamase-1-producing Raoultella planticola. Microb Drug Resist 2017; 23: 127–131. 27754764 10.1089/mdr.2015.0346 PMC5206690

[17] Hsu PI Tsai FW Kao SS Hsu WH Cheng JS Peng NJ Ten-day quadruple therapy comprising proton pump inhibitor, bismuth, tetracycline, and levofloxacin is more effective than standard levofloxacin triple therapy in the second-line treatment of *Helicobacter pylori* infection: A randomized controlled trial. Am J Gastroenterol 2017; 112: 1374–1381. 28719592 10.1038/ajg.2017.195

[18] Humphries RM Hindler JA Shaffer K Campeau SA. Evaluation of ciprofloxacin and levofloxacin disk diffusion and Etest using the 2019 *Enterobacteriaceae* CLSI breakpoints. J Clin Microbiol 2019; 57: e01797–18. 30567744 10.1128/JCM.01797-18 PMC6425192

[19] Ismail SJ Mahmoud SS. First detection of New Delhi metallo-β-lactamases variants (NDM-1, NDM-2) among *Pseudomonas aeruginosa* isolated from Iraqi hospitals. Iran J Microbiol 2018; 10: 98–103. 29997749 PMC6039455

[20] Asif M. Role of quinolones and quinoxaline derivatives in the advancement of treatment of tuberculosis. Int J Sci World 2015; 3: 18–36.

[21] Garoff L Yadav K Hughes D. Increased expression of Qnr is sufficient to confer clinical resistance to ciprofloxacin in *Escherichia coli*. J Antimicrob Chemother 2018; 73: 348–352. 29106520 10.1093/jac/dkx375 PMC5890660

[22] Kusachi S Oe K Okuda Y Sasaki J Maetani I Watanabe M Clinical study on levofloxacin injection for surgical infection. Japan J Chemother 2017; 65: 445–455.

[23] Noel GJ. A review of levofloxacin for the treatment of bacterial infections. Clin Med Ther 2009; 1: 433–458.

[24] Wu H-H Liu H-Y Lin Y-C Hsueh P-R Lee Y-J. Correlation between levofloxacin consumption and the incidence of nosocomial infections due to fluoroquinolone-resistant *Escherichia coli*. J Microbiol Immunol Infect 2016; 49: 424–429. 22560475 10.1016/j.jmii.2011.12.019

[25] Bax BD Chan PF Eggleston DS Fosberry A Gentry DR Gorrec F Type IIA topoisomerase inhibition by a new class of antibacterial agents. Nature 2010; 466: 935–940. 20686482 10.1038/nature09197

[26] Iredell J Brown J Tagg K. Antibiotic resistance in Enterobacteriaceae: mechanisms and clinical implications. BMJ 2016; 352:h6420. 26858245 10.1136/bmj.h6420

[27] Aldred KJ Kerns RJ Osheroff N. Mechanism of quinolone action and resistance. Biochemistry 2014; 53: 1565–1574. 24576155 10.1021/bi5000564 PMC3985860

[28] Cormier R Burda WN Harrington L Edlinger J Kodigepalli KM Thomas J Studies on the antimicrobial properties of N-acylated ciprofloxacins. Bioorg Med Chem Lett 2012; 22: 6513–6520. 22995622 10.1016/j.bmcl.2012.05.026 PMC3757340

[29] Collin F Karkare S Maxwell A. Exploiting bacterial DNA gyrase as a drug target: current state and perspectives. Appl Microbiol Biotechnol 2011; 92: 479–497. 21904817 10.1007/s00253-011-3557-z PMC3189412

[30] Ahmed A Daneshtalab M. Nonclassical biological activities of quinolone derivatives. J Pharm Pharm Sci 2012; 15: 52–72. 22365088 10.18433/j3302n

[31] Wu H-H Liu H-Y Lin Y-C Hsueh P-R Lee Y-J. Correlation between levofloxacin consumption and the incidence of nosocomial infections due to fluoroquinolone-resistant *Escherichia coli*. J Microbiol Immunol Infect 2016; 49: 424–429. 22560475 10.1016/j.jmii.2011.12.019

[32] Zhanel GG Hartel E Adam H Zelenitsky S Zhanel MA Golden A Solithromycin: a novel fluoroketolide for the treatment of community-acquired bacterial pneumonia. Drugs 2016; 76: 1737–1757. 27909995 10.1007/s40265-016-0667-z

[33] Dalhoff A. Global fluoroquinolone resistance epidemiology and implictions for clinical use. Interdiscip Perspect Infect Dis 2012; 2012: 976273. 23097666 10.1155/2012/976273 PMC3477668

[34] Lee S-J Lee DS Choe HS Shim BS Kim CS Kim ME Antimicrobial resistance in community-acquired urinary tract infections: results from the Korean antimicrobial resistance monitoring system. J Infect Chemother 2011; 17: 440–446. 21140281 10.1007/s10156-010-0178-x

[35] Akram M Shahid M Khan AU. Etiology and antibiotic resistance patterns of community-acquired urinary tract infections in JNMC Hospital Aligarh, India. Ann Clin Microbiol Antimicrob 2007; 6: 4. 17378940 10.1186/1476-0711-6-4 PMC1852324

[36] Yassine I Rafei R Osman M Mallat H Dabboussi F Hamze M. Plasmid-mediated quinolone resistance: Mechanisms, detection, and epidemiology in the Arab countries. Infect Genet Evol 2019; 76:104020. 31493557 10.1016/j.meegid.2019.104020

[37] Yokota S-i Sato T Okubo T Ohkoshi Y Okabayashi T Kuwahara O Prevalence of fluoroquinolone-resistant *Escherichia coli* O25: H4-ST131 (CTX-M-15-non-producing) strains isolated in Japan. Chemotherapy 2012; 58: 52–59. 22343392 10.1159/000336129

[38] Jang WH Yoo DH Park SW. Prevalence of and risk factors for levofloxacin-resistant *E. coli* isolated from outpatients with urinary tract infection. Korean J Urol 2011; 52: 554–559. 21927703 10.4111/kju.2011.52.8.554 PMC3162222

[39] Hsueh P-R Hoban DJ Carmeli Y Chen S-Y Desikan S Alejandria M Consensus review of the epidemiology and appropriate antimicrobial therapy of complicated urinary tract infections in Asia-Pacific region. J Infect 2011; 63: 114–123. 21669223 10.1016/j.jinf.2011.05.015

[40] Bengtsson-Palme J Kristiansson E Larsson DJ. Environmental factors influencing the development and spread of antibiotic resistance. FEMS Microbiol Rev 2018; 42: fux053. 10.1093/femsre/fux053 PMC5812547 29069382

[41] Ventola CL. The antibiotic resistance crisis: part 1: causes and threats. P T 2015; 40: 277–283. 25859123 PMC4378521

[42] Gharagozloo R Ghavamian P. Bacteriological survey of urinary tract infection. Acta Med Iran 1968; 3–4: 105–119.

[43] Haeili MGA Nomanpour B Omrani M Feizabadi MM. Drug resistance patterns of bacteria isolated from patients with nosocomial pneumonia at Tehran hospitals during 2009–2011. J Infect Dev Ctries 2013; 7: 312–7. 23592640 10.3855/jidc.2604

[44] Akya A Chegenelorestani R Elahi A Hamzavi Y. Frequency of plasmid-mediated quinolone resistance genes in extended-spectum β-lactamase-producing *Escherichia coli*. J Mazandaran Univ Med Sci 2017; 27: 41–51.

[45] Leylabadlo HEEH Pourlak T Aghazadeh M Kafil HS Samadi H. Extended-spectrum beta-lactamase producing Gram negative bacteria in Iran. Afr J Infect Dis 2017; 11: 39–53. 28670639 10.21010/ajid.v11i2.6 PMC5476812

[46] Firoozeh F Zibaei M Soleimani-Asl Y. Detection of plasmid-mediated qnr genes among the quinolone-resistant *Escherichia coli* isolates in Iran. J Infect Dev Ctries 2014; 8: 818–822. 25022290 10.3855/jidc.3746

[47] Rezazadeh M Baghchesaraei H Peymani A. Plasmid-mediated quinolone-resistance *(qnr)* genes in clinical isolates of *Escherichia coli* collected from several hospitals of Qazvin and Zanjan provinces, Iran. Osong Public Health Res Perspect 2016; 7: 307–312. 27812489 10.1016/j.phrp.2016.08.003 PMC5079201

[48] Rashki A Rahdar M Ghalehnoo ZR. Characterization of uropathogenic *Escherichia coli*: Distribution of adhesin-encoding genes and O-serotypes among ciprofloxacin susceptible and resistant isolates. Jundishapur J Microbiol 2019; 12(9):e89179.

[49] Iranpour D Hassanpour M Ansari H Tajbakhsh S Khamisipour G Najafi A. Phylogenetic groups of *Escherichia coli* strains from patients with urinary tract infection in Iran based on the new Clermont phylotyping method. Biomed Res Int 2015; 2015: 846219. 25692147 10.1155/2015/846219 PMC4322292

[50] Malekzadegan Y Rastegar E Moradi M Heidari H Sedigh Ebrahim-Saraie H. Prevalence of quinolone-resistant uropathogenic *Escherichia coli* in a tertiary care hospital in south Iran. Infect Drug Resist 2019; 12: 1683–1689. 31354317 10.2147/IDR.S206966 PMC6590898

[51] Abbasi P Kargar M Doosti A Mardaneh J Ghorbani-Dalini S Dehyadegari MA. Molecular detection of diffusely adherent *Escherichia coli* strains associated with diarrhea in Shiraz, Iran. Arch Pediatr Infect Dis 2017; 5 (2); e37629.

[52] Ghorbani-Dalini S Kargar M Doosti A Abbasi P Sarshar M. Molecular epidemiology of ESBL genes and multi-drug resistance in diarrheagenic *Escherichia coli* strains isolated from adults in Iran. Iran J Pharm Res 2015; 14: 1257–1262. 26664394 PMC4673955

[53] Kazemian H Heidari H Ghanavati R Mohebi R Ghafourian S Shavalipour A Characterization of antimicrobial resistance pattern and molecular analysis among extended spectrum β-Lactamase-producing *Escherichia coli*. Pharm Sci 2016; 22: 279–284.

[54] Pajand O Ghassemi K Kamali F Taghavipoor S Hojabri Z. Investigation of phylogenetic diversity among *Eschereshia coli* isolates recovered from hospitalized patients. Koomesh 2017; 19: 207–212.

[55] Azimi T Maham S Fallah F Azimi L Gholinejad Z. Evaluating the antimicrobial resistance patterns among major bacterial pathogens isolated from clinical specimens taken from patients in Mofid Children’s Hospital, Tehran, Iran: 2013–2018. Infect Drug Resist 2019; 12: 2089–2102. 31410032 10.2147/IDR.S215329 PMC6645606

[56] Mirzaii M Jamshidi S Zamanzadeh M Marashifard M Hosseini SAAM Haeili M Determination of *gyrA* and *parC* mutations and prevalence of plasmid-mediated quinolone resistance genes in *Escherichia coli* and *Klebsiella pneumoniae* isolated from patients with urinary tract infection in Iran. J Glob Antimicrob Resist 2018;13:197–200. 29747008 10.1016/j.jgar.2018.04.017

[57] Manyi-Loh C Mamphweli S Meyer E Okoh A. Antibiotic use in agriculture and its consequential resistance in environmental sources: potential public health implications. Molecules 2018; 23: 795. 10.3390/molecules23040795 PMC6017557 29601469

